# Reported Barriers to Hepatitis C Treatment among Pregnant and Early-Parenting Mothers Undergoing Substance Use Disorder Treatment in One U.S. State

**DOI:** 10.3390/idr14010001

**Published:** 2021-12-22

**Authors:** Ayooluwatomiwa Deborah Adekunle, Kathi L. Harp, Zaynab G. Al-Abdali, Agatha S. Critchfield, Sheila Barnhart, Kathleen T. Winter

**Affiliations:** 1 Division of Internal Medicine, St. Luke’s Hospital, Chesterfield, MO 63017, USA; 2 College of Public Health, University of Kentucky, Lexington, KY 40508, USA; Kathi.harp@uky.edu (K.L.H.); zaynab.abdali@gmail.com (Z.G.A.-A.); Kathleen.winter@uky.edu (K.T.W.); 3 Baptist Health Medical Group, Lexington, KY 40503, USA; agatha.s.critchfield@gmail.com; 4 College of Social Work, University of Kentucky, Lexington, KY 40508, USA; Sheila.barnhart@uky.edu

**Keywords:** hepatitis C, HCV, substance use disorder and treatment, treatment barriers, vulnerable populations, co-occurring SUD and HCV

## Abstract

Nationwide, the prevalence of the hepatitis C virus (HCV) has risen in recent years. At least 90% of infected persons must be treated to achieve global elimination targets. The current study aimed to explore barriers to, and facilitators of, direct-acting antiviral (DAA) HCV treatment uptake amongst pregnant and early-parenting women undergoing comprehensive substance use treatment. Twenty participants with documented HCV antibody positivity were recruited from two substance use treatment centers in central Kentucky. Semi-structured interviews were conducted to explore knowledge about HCV, previous experiences, and intentions to seek care. Themes were extracted using an inductive analytical approach. Most participants were aware of the dangers posed by HCV infection. However, there was a high degree of misinformation about transmission mechanisms and treatment eligibility requirements. Low priority for HCV treatment also surfaced as a barrier to treatment uptake. Participants reported being unable to seek care due to time and resource limitations in the presence of a highly demanding treatment process. Findings from the current study suggest that more work is needed to eliminate residual barriers that limit access to HCV treatment among pregnant and early-parenting women in treatment for substance use disorder.

## 1. Introduction

The increase in hepatitis C (HCV) incidence in the United States in recent years is largely attributed to the opioid epidemic and has predominantly affected younger Americans between 18–39 years of age [1]. Among women of reproductive age, HCV antibody seropositivity rates increased approximately 100% between 2006 and 2014, creating an increased potential for mother-to-child transmission [2]. Vertical transmission represents the most common cause of pediatric HCV in the United States [3].

An estimated 60–85% of HCV-infected persons will progress to chronic infection [4], which accounts for over 350,000 annual deaths and, globally, is the leading cause of end-stage liver disease requiring liver transplant [5,6]. The factors that predispose one to chronicity are not fully understood; however, with appropriate therapy, a cure can be achieved [7].

Experts estimate that at least 90% of the HCV-infected population needs to be diagnosed and treated to achieve the World Health Organization’s 2030 elimination targets [8,9]. As of June 2018, only 12 countries were on track, and the United States was not one of them [10]. COVID-19 has further compounded the problem as it has led to decreases in prescriptions for HCV treatment medications during the pandemic [11].

Prior to the introduction of direct-acting antiviral (DAA) agents, HCV treatments involved interferon-based regimens which had many undesirable side effects, required long durations of treatment, and only resulted in sustained virological response (SVR) in about 50% of patients [12,13]. In contrast, with currently available DAA treatments, SVR is now achievable in over 90% of patients using an oral regimen over 8–12 weeks, with fewer side effects than interferons [14].

For every one symptomatic case reported, 13.9 new HCV infections are estimated to occur [15]. Many diagnosed cases go untreated [16]. Pregnancy presents an opportunity for testing and engaging HCV-infected women into care because women often have insurance coverage during pregnancy and are typically in contact with the health care system during the perinatal period [17]. Additionally, pregnancy often provides the needed motivation to take beneficial actions for one’s health [18,19]. Although HCV treatment during pregnancy is not recommended, successful treatment after delivery will prevent perinatal transmission in future pregnancies.

Incorporating HCV DAA treatment into substance use treatment and harm reduction centers has been shown to result in higher HCV treatment initiation and completion rates [20,21]; however, this model of care has yet to be widely implemented.

### Barriers to Treatment Uptake

Eligibility restrictions on insurance coverage for HCV treatment have largely hindered optimal DAA uptake. Before 2018, Kentucky Medicaid restricted DAA treatment coverage to individuals with advanced-stage liver fibrosis and who had met certain minimum sobriety requirements [22]. Consequently, in 2016, less than 0.03% of Kentucky’s Medicaid-insured HCV-infected patients received treatment [23]. In 2018, coverage eligibility for Kentucky Medicaid expanded in line with the American Association for the Study of Liver Diseases (AASLD) and Infectious Diseases Society of America’s (IDSA) recommendations [17,22]. Data are limited for Kentucky, but a study conducted in Oregon revealed that, although elimination of treatment restrictions increased treatment among previously ineligible individuals in the year after restrictions were lifted, it did not necessarily result in a sustained increase in treatment uptake over time [24].

It is unclear why DAA uptake remains sub-optimal despite expanded eligibility. Studies examining determinants of uptake among people who inject drugs (PWIDs) found a mix of factors, including poor vein health, lack of symptoms, procrastination, and continued illicit drug use, to be key players in poor uptake [25,26]. HIV co-infection and concern from loved ones have also been found to facilitate treatment uptake [26].

In the presence of expanded eligibility and safer pharmacological agents, it is imperative to seek to eliminate residual barriers to treatment uptake. We evaluated barriers to and facilitators of DAA uptake among HCV-infected pregnant and early-parenting women receiving comprehensive treatment for substance use disorder (SUD). An increased ability to engage this target population with treatment would result in not only a reduction of HCV prevalence, but also the prevention of vertical transmission in future pregnancies.

## 2. Methods and Materials

The study sample was comprised of 20 women with opioid use disorder (OUD) receiving care at two comprehensive substance use treatment (CSUT) centers in central Kentucky. These facilities specialize in SUD treatment of pregnant and early-parenting women. As part of routine clinical care, patients with OUD who are receiving medication-assisted therapy (MAT) are screened for prior or current HCV infection, and all eligible patients with antibodies to HCV were invited to complete an iPad survey about their knowledge, attitudes, and experiences regarding HCV transmission and treatment. Written informed consent was obtained from study participants. Women who were incarcerated, had serious mental health problems, could not be located, were younger than 18 years of age, or did not consent to the study were excluded. Forty-five of 63 eligible women completed the initial survey, resulting in a 71.4% response rate. The initial survey included an item asking if participants would like to be contacted about participating in a follow-up study where they would be interviewed to learn more in-depth information about their HCV knowledge, attitudes, and experiences. Of the 45 women who completed the initial survey, 20 (44.4%; five pregnant and 15 early-parenting) were available and gave consent to complete in-depth follow-up interviews. These respondents included pregnant and non-pregnant women who reported previously seeking treatment for HCV, as well as those who reported never seeking treatment.

Interviews were conducted during routine clinic visits over a 10-week period. Participants were given a $10 baby item for their participation. Interviews were performed by two trained research assistants who were familiar with current HCV treatment protocols and policy changes related to HCV treatment in Kentucky. Prior to the qualitative interviews, they built rapport and trust with participants through a series of informal interactions. Interviews were conducted within the treatment facilities, which have been cultivated as safe, judgement-free environments. A semi-structured guide was used to direct the interview process and ensure uniformity. When indicated, interviewers were able to probe interviewees for additional information or ask for clarification on previous responses. The interviews explored time of HCV diagnosis, reaction to diagnosis, perceptions about how they contracted HCV, status disclosure, prior experiences with treatment, plans regarding future treatment, and barriers to and facilitators of treatment uptake. Barriers to treatment uptake were explored in terms of participants’ experience and perception. Experience was framed as “based on personal experience”, while perception was framed as “based on mind constructed models of the world which may or may not have been personally experienced” [27].

Interviews were recorded using a Yemenren R9 audio recording device and transferred to a secure network file to ensure confidentiality. Three interview audio files became corrupted during the process of file transfer, resulting in a total of 17 usable interview recordings and a final sample size of 17 women (four pregnant and 13 early parenting).

Demographic information was extracted from participants’ medical records and matched with their survey and interview responses.

Audio files were transcribed verbatim and identifying information, such as provider or participant name, was redacted. An inductive analytical approach was used to analyze interview transcripts. This approach is often used in qualitative studies to condense text data into summarized formats by repeatedly evaluating the data to identify emerging themes. Two independent raters familiarized themselves with the data by reading and re-reading the interview transcripts to identify key themes. Open coding with note taking was then conducted. After this initial process, the data were then grouped and regrouped based on the coding frame to establish broad categories of perceived and experienced barriers to and facilitators of DAA uptake, as well as broad categories of responses to diagnoses. Broad categories were discussed by both raters and a 100% agreement was reached between them. Each theme is supported by quotes that have been included in the results section. The study was approved by The University of Kentucky’s Institutional Review Board (IRB approval number: 45039).

## 3. Results

The sample included 17 women, four pregnant and 13 early-parenting, who ranged from 22–42 years of age. Among participants, four had spontaneously cleared the HCV virus and two had been successfully treated at the time of interview (Table 1). Participants described responses to their diagnoses that included distress, not being surprised, fear, and, in one instance, shock. Although most patients connected their HCV infection to injection drug use, some did not understand the mechanism by which transmission occurred and reported giving it to themselves by reusing their own needles.

“I thought that I got it from my baby dad but then I found out that I could give it to myself from using dirty needles.” Postnatal, 31.

“I figured I probably gave it to myself because I used needles on myself.” Postnatal, 33.

Through a series of questions exploring previously experienced and current factors affecting treatment uptake, several facilitators and barriers emerged.

### 3.1. Barriers to Treatment Uptake

#### 3.1.1. Low Priority Due to Other Life Circumstances

In response to the question, “What factors have made it difficult for you to get HCV treatment?”, a number of responses were elicited, ranging from a busy schedule to lack of transportation to simply forgetting (Table S1).

“Well, I really don’t think about it because I got some ‘shit’ going on.” Postnatal, 31.

“I have been homeless, so I just rent a lone room in a place. I just, I have been, I’m not that settled. So, to make appointments and I work weekends and I work night shifts, So I just don’t got time, right now to.” Pregnant, 42.

Another participant said transportation and work could prevent her from seeking treatment.

“Just like transportation and work.” Postnatal, 32.

“I kinda forgot about it.” Postnatal, 30.

These and other responses highlight that, for some respondents, HCV treatment is not viewed as a high priority. In some cases, this is because of competing life demands, while in others, getting treatment is not viewed as pertinent to health and was forgotten about altogether.

#### 3.1.2. Uncertainty about Currently Available Treatments and Side Effects

Perceptions about medication side effects were reported as a barrier to treatment uptake.

“I think it’s the medicine, like being scared of the medicine and the side effects because that’s what it was with me. Like they told me I would be sick, they told me I had to eat this much and I would feel really down and low and they had me scared and now people are saying it’s not going to make you feel like that. I don’t know, I guess I’ll find out…” Postnatal, 33.

“No just knowing that it’s not that interferon. You know for a while, that was the only option. If it was still just that, I don’t know that I would do it but now that there is more than just that, I don’t have any problem with getting treatment.” Postnatal, 31.

The potential for experiencing severe side effects made patients less inclined to seek treatment. This finding underscores the importance of providing accurate information regarding the improved tolerability of newer medicines.

#### 3.1.3. Uncertainty/Misinformation about Treatment Eligibility Requirements

For some women, the perception of treatment ineligibility due to insurance policy restrictions was a major barrier, while others had received inconsistent information from healthcare providers about the necessary criteria needed to obtain treatment. One interviewee reported having been turned away multiple times, while others reported believing they would be eligible for treatment only after attaining a certain amount of what they described as “clean time” (extended period of abstinence from drugs).

“… every time I’ve come back and it can’t happen for some reason like, I was dirty, I wasn’t clean long enough, I was on suboxone for too long, I was pregnant. So, hopefully this time would be a little different.” Pregnant, 25.

“I’ve always been told that you have to be clean for six months before your insurance will pay for it and I just hit six months…” Postnatal, 31.

“You have to have a certain amount of clean time- I’m not sure what it is but I know I’ve hit it.” Postnatal, 30.

“Yes, after six months of my treatment and I’ve got that now.” Postnatal, 22.

These responses demonstrate how previous insurance coverage restrictions, although now removed, continue to contribute to low treatment uptake. They also suggest that merely removing insurer restrictions does not suffice and further efforts to create awareness about eligibility policy changes and reengage previously turned away patients (when possible) are warranted.

#### 3.1.4. Active Substance Use

The theme of ongoing substance use emerged when participants were asked about potential barriers to HCV treatment uptake not for themselves, but for other similarly situated women.

“… When you’re in the ‘madness’, you know, you’re not going to worry about treating something that you got from using a dirty needle when you’re still using a dirty needle… Again, in the highness, you don’t, you’re not worried about Hep C. You might talk about it before you use a needle after somebody like, ‘You don’t have Hep C, do ya?’, but that’s about it.” Postnatal, 31.

“… wrapped up in drug use, just not taking the time to take care of their bodies like they should.” Postnatal, 41.

“Know when you are active in addiction, you don’t, that’s the last thing on your mind. You’ve got to get your fix because that’s the first thing, you know that’s what heals you in the moment.” Postnatal, 36.

“They still want to use.” Postnatal, 32.

These women view ongoing substance use as an impediment to proper self-care and, consequently, treatment uptake.

#### 3.1.5. Stigma

Although no study participant reported stigma as a personal barrier to seeking care, some reported not disclosing their HCV status for fear of being stigmatized. One person even reported this fear of stigma as her motivation to seek care.

“I don’t tell anybody because people do judge but no I sought care so that I could get rid of it so that I would not have to tell the community if I got hurt.” Postnatal, 32.

Others, however, reported perceiving stigma surrounding HCV as a barrier that could prevent infected people from seeking treatment.

“Fear that they might, people might know that they have it.“ Pregnant, 31.

“Being treated differently. People looking at them differently.“ Pregnant, 41.

“Scared of having it, what that means. Like a lot of people don’t realize that a lot of it is just cirrhosis of the liver. Alcoholics have cirrhosis of the liver and that’s what Hep C causes. I think it’s the stigma behind it.” Postnatal, 32.

These women believe that, for some in their situation, the cost of their HCV statuses potentially becoming publicized outweighed the benefit of seeking treatment and the potential to be cured.

#### 3.1.6. Complexity of the Treatment Process

The complicated uptake process involving repeated hospital appointments was also perceived as a barrier to treatment uptake.

“You are going to have to get in a doctor, see them and they have to refer you to the liver specialist. It’s just a lot of steps to get you there and it’s worth it but if you are not, you’ve got to be committed to get it done because you can’t miss any appointments, you’ve got to go get blood drawn every two weeks. It’s a lot. And if you don’t have a car, it’s like how the hell are you going to get there?” Postnatal, 31.

“If you don’t have the medical care or access to it as well or transportation—I mean it’s a lot.” Postnatal, 32.

These responses, among others, suggest that individuals with complex healthcare needs (in this case, OUD and HCV) would likely benefit from a system that is better suited to meet their needs.

### 3.2. Facilitators of Treatment Uptake

As with barriers, facilitators of treatment uptake were explored both in terms of personal experience, as well as perceptions about treatment facilitators experienced by others. Participants reported various forms of support as factors that could facilitate treatment uptake.

“If someone would give me money for the day of work I miss. That would probably be it [motivation to initiate treatment].” Pregnant, 42.

“I do worry about being able to take the other one like at the same time, all the time, like that might be rough but I think doing it at the recovery house, I think that’s a good way to start because I mean, your medicine is monitored, you have to go get it from somebody else. I feel like now is probably the best time to start it, before I’m on my own.” Postnatal, 31.

#### Provision of Information

The need for more and better information was an almost universal response to a question about what factors participants perceived would make treatment uptake easier for other similarly situated people. Participants reported perceiving a scarcity of information regarding HCV infection. One participant even described her primary care provider’s unwillingness to engage with her HCV infection.

“I don’t know, maybe just get it out there to where people actually can hear that there is a treatment and because a lot of people are worried about insurance this, insurance that, just let them know that no, they can get it because it’s there. It surprised me that it’s there.” Postnatal, 33.

“I have only ever heard Hep C talked about in detox or centers like this. I’ve never heard a regular doctor or anyone else. Even when I went to my PCP and said I am trying to get treated, he never, he never, he wasn’t gonna touch it. Making information available if you’re not in treatment or around detox. And letting people know that the treatment is not like chemo.” Postnatal, 30.

“I didn’t know the regulations have changed. A lot of people don’t know. They still feel like you have to be in treatment two to three years before you can get it. I think there should be more information put out there that there is more access to it with less restrictions right now.” Postnatal, 32.

“Just more information like, not knowing if you have to be clean for so long or not knowing what the options are. I never see anything about the treatment like you see a lot of stuff about, do you have Hep C?” Postnatal, 31.

These women assert that there is a need to get the word out about HCV infection, the available treatment modalities, and insurance eligibility requirements in order to increase treatment uptake.

## 4. Discussion

This study explored facilitators of, and barriers to, DAA uptake for HCV among a sample of women receiving comprehensive SUD treatment, including MAT, for OUD. The findings highlight that, although policy changes have been implemented to improve access to HCV treatment, residual barriers exist that prevent optimal uptake.

Comparable to findings in a similar study [25], patients reported gaps in provider communication regarding care for their HCV. In cases where initial information had been given to the women in our study, they didn’t receive updated information when, for example, policy changes were implemented that expanded insurance coverage and eligibility. Patients reported believing that they would be subject to sobriety requirements, which have been eliminated under newer policies. They also reported concerns about medication side effects, which are known to be significantly lower with DAAs [12,13]. These findings underscore the importance of effective communication and patient education in healthcare, especially in the era of a multiplicity of health information sources [28]. Not only does effective patient education help combat misperceptions, but it can also empower patients with knowledge and motivate them to initiate treatment [29,30]. Clinicians play a critical role in ensuring that HCV-infected patients in their care are adequately informed about policy changes pertaining to treatment. Harm reduction centers, which may be the only contact some patients have with the health care system, could also play an important role in information dissemination. Finally, social media is a currently untapped resource; as a common source of information in this era, it could be utilized effectively as a tool to combat misperceptions and provide information [31]. The media could also undertake efforts to reduce the stigma associated with HCV by using targeted messaging, as was done for HIV in the late 1990s through early 2000s [32].

Restrictions surrounding prescriber permissions have contributed greatly to the complexity of DAA uptake. Participants in the current study described having to go through multiple steps, including first getting a primary care provider (PCP), and then obtaining a referral from that PCP to see a subspecialist who would eventually administer treatment. Replacing this long and drawn-out process with one that provides HCV treatment at the primary care level could substantially improve treatment uptake [33,34]. Achieving this in the United States would require stakeholder engagement to eliminate insurer-imposed prescriber restrictions and increase provider buy-in. Training and resource provision for clinicians with no prior HCV treatment experience would also be necessary [35]. The introduction of project Extension for Community Healthcare Outcomes (ECHO) has begun to partly address this problem by allowing front-line clinicians to receive the required support from specialist mentors via telehealth [36,37]. Research has shown this method of care delivery to be non-inferior to specialist care in achieving sustained virological response (SVR) among patients receiving treatment for HCV [38]. With the expansion of telehealth availability due to the COVID-19 pandemic [39], opportunities to engage patients with HCV in DAA treatment through telemedicine could significantly improve rates of treatment uptake and completion by eliminating the time- and transportation-related barriers cited by many of the women we interviewed.

Individuals with substance use disorders are often affected by low socioeconomic status, unstable employment, and housing, and depend on public services [40,41]. Participants in our study described lacking access to transportation and, in one instance, the inability to take time off work (for HCV treatment) after just having gone through a period of homelessness. Such experiences highlight the importance of incorporating HCV treatment into existing healthcare visits, as well as expanding telehealth treatment of HCV in order to minimize the burden of additional appointments. At the time of writing, this is being implemented at both study sites.

Other forms of support can assist with HCV treatment adherence. DAA therapy requires the consumption of one to three pills per day for 8–12 weeks [35]. This treatment could be burdensome for some patients and mechanisms could be put in place to assist patients who perceive a lack of self-efficacy to successfully adhere to the regimen. Some mechanisms aimed at improving treatment adherence that have been used for other health conditions include mobile phone SMS reminders [42], peer assistance, and direct observation [43]. Treatment adherence improvement strategies, when contextually appropriate, could prove beneficial for women like those in our study who are pregnant/early-parenting and have multiple health conditions for which they need care.

While some interviewees perceived ongoing drug use as a barrier to HCV treatment initiation, another study involving participants with HCV enrolled in a care coordination program found no difference in SVR between patients receiving MAT for co-occurring SUD and those who were actively using drugs [44]. Other studies have found no correlation between drug use and treatment adherence, attendance for follow-up, and attainment of SVR [45,46,47]. These findings suggest that, with appropriate measures in place, health care systems can successfully engage HCV-infected individuals with comorbid substance use disorders with care.

Comparable to findings in a separate study, participants reported a perception of judgement and stigma as a potential deterrent to seeking care, while reporting a judgement-free environment as a facilitator [25]. Stigma has been shown to impede successful health care delivery [48]. It is crucial that health care systems implement strategies to reduce all forms of stigma and judgement.

A key limitation to this study is the loss of three audio files that became corrupted during transfer from the recording device to a secure computer network, although the interviewers noted they did not believe the contents of those files distinctively differed from the intact files based on their recollections from the interviews. Another limitation was that all members of the target population were pregnant or early-parenting women receiving MAT who were already in regular contact with the healthcare system; thus, we were unable to capture perspectives from similarly situated women not in regular contact with the health care system. Additionally, our interviews did not explore information-seeking behaviors which could provide insight into potential strategies for combatting misinformation. Furthermore, this study was exploratory in nature and, due to the very small sample size, its findings are not generalizable to the larger population of pregnant/early-parenting women with co-occurring HCV and OUD. Rather, our findings are intended to be used as hypothesis-generating to inform future research investigations on this topic. The authors note the qualitative nature of this study as a major offsetting strength because it allowed for in-depth exploration and probing to elicit more detailed responses from interviewees.

## 5. Conclusions

This study explored residual barriers to and facilitators of DAA uptake among a sample of pregnant/early-parenting women with HCV receiving treatment (and MAT) for OUD. Misinformation, the difficulty of navigating an often-complicated treatment process, and the perception of stigma emerged as barriers to treatment uptake, while various forms of support and information about the treatment process and eligibility guidelines were viewed as potential facilitators for treatment uptake. Further studies building on these findings, particularly among similarly complex women not in regular contact with the healthcare system, are encouraged.

## Figures and Tables

**Table 1:** Characteristics of study participants.

Category	Age	Clinical Status
Early-parenting	33	Infected
Early-parenting	30	Infected
Early-parenting	31	infected
Early-parenting	31	Cleared
Pregnant	41	Infected
Pregnant	25	Infected
Early-parenting	31	Successfully treated
Early-parenting	27	Cleared
Early-parenting	36	Infected
Pregnant	31	Infected
Early-parenting	32	Successfully treated
Pregnant	42	Cleared
Early-parenting	31	Infected
Early-parenting	31	Infected
Early-parenting	35	Infected
Early-parenting	22	Cleared
Early-parenting	35	Infected

## Data Availability

Given the sensitivity of the subject matter, only redacted versions of the transcripts can be provided upon request. Please email all requests to Deborah.adekunle@uky.edu.

## Supplementary Materials

The following are available online at https://www.mdpi.com/article/10.3390/idr14010001/s1, Table S1: Summary of Reported HCV Treatment Barriers and Facilitators among a Sample of Pregnant/Early Parenting Mothers in SUD Treatment.
