# Supplementary material for: Reported Barriers to Hepatitis C Treatment among Pregnant and Early-Parenting Mothers Undergoing Substance Use Disorder Treatment in One U.S. State
Source: Infect Dis Rep. 2021 Dec 22;14(1):1–11. doi: 10.3390/idr14010001 (PMC8788261; doi:10.3390/idr14010001)
Supplement: Supplementary file 1 [file idr-14-00001-s001.zip › idr-1470034-supplementary.pdf]

Table S1: Summary of Reported HCV Treatment Barriers and Facilitators among a Sample of Pregnant/Early Parenting Mothers in SUD Treatment

**Category**	**Themes**
Barriers to treatment uptake	• Low priority for treatment due to other life circumstances<br>• Uncertain/ misinformed about:<br>○ Treatment eligibility<br>○ Treatment duration and/or side effects<br>• Active/ongoing substance use<br>• Perceived complexity of the treatment process
Facilitators of treatment uptake*	• Provision of information by healthcare providers<br>• Support to initiate and complete treatment:<br>○ Logistic support<br>○ Financial support

*Given the low prevalence of treatment uptake among our study population, facilitators of treatment uptake could only be explored hypothetically.
